# Metoprolol Protects Against Arginine Vasopressin-Induced Cellular Senescence in H9C2 Cardiomyocytes by Regulating the Sirt1/p53/p21 Axis

**DOI:** 10.1007/s12012-021-09704-8

**Published:** 2021-11-20

**Authors:** Qiang Li, Kang Huang, Tianyi Ma, Shijuan Lu, Shilin Tang, Miao Wu, Hui Yang, Jianghua Zhong

**Affiliations:** Department of Cardiology, Haikou Affiliated Hospital of Central South University Xiangya School of Medicine, No. 43 Renmin Avenue, Meilan District, Haikou, 570208 Hainan China

**Keywords:** Metoprolol, Cardiac hypertrophy, p53, Cell senescence

## Abstract

Cardiomyocyte senescence is involved in the pathological mechanism of cardiac diseases. Metoprolol is a β1 receptor blocker used for the treatment of hypertension. Recent studies show that Metoprolol can protect cardiomyocytes against ischemia injury. The present study aims to investigate the protective effects of Metoprolol against arginine vasopressin (AVP)-induced cellular senescence in cultured cardiomyocytes. The cell proliferation assay and cytotoxicity lactate dehydrogenase assay showed that the highest tolerated dosage of Metoprolol in H9C2 cardiomyocytes was optimized as 10 µM. The enzyme-linked immunosorbent assay showed that Metoprolol significantly ameliorated the elevated level of the DNA oxidation product 8-hydroxy-2 deoxyguanosine. Metoprolol also decreased the percentage of senescence-associated β-galactosidase positive cells and improved the telomerase activity under AVP exposure. Moreover, treatment with Metoprolol ameliorated the decreased intracellular nicotinamide phosphoribosyltransferase activity, nicotinamide adenine dinucleotide/nicotinamide adenine dinucleotide phosphate (NAD^+^/NADPH) ratio, and Sirtuin1 activity in cardiomyocytes by AVP. Finally, Metoprolol was able to downregulate the AVP-induced expression of acetylated p53 and p21. Taken together, our data reveal that Metoprolol protected the cardiomyocytes from AVP-induced senescence.

## Introduction

**Fig. 1:**
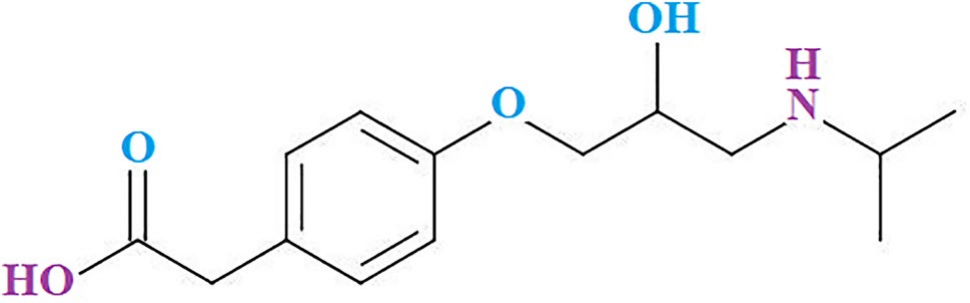
Molecular structure of Metoprolol

Pathological cardiac hypertrophy is a compensatory mechanism by which cardiomyocytes adapt to stress overload, which is a high risk for the pathogenesis of the decline of left ventricular function and chronic heart failure [1, 2]. The clinical trials show that cardiac hypertrophy is the independent risk factor for disability and fatality rates induced by cardiac disease [3]. It is of high interest to patient care to investigate the underlying pathological mechanism of cardiac hypertrophy. Recent studies find that cardiomyocyte senescence is involved in the pathogenesis of pathological cardiac hypertrophy [4]. Cell senescence is a state of cellular proliferative arrest induced by several internal and external elements, such as oxidative stress and inflammation [5] and is mainly characterized by an increased cell size, promoted senescence-associated β-galactosidase (SA-β-gal) activity, and upregulated cyclin-dependent kinase inhibitors (CDKIs), such as p53, p21, and p16 [6, 7]. Compared to apoptosis, senescent cells maintain a certain metabolic activity and impact the surrounding microenvironment by secreting multiple types of growth factors, cytokines, inflammatory factors, and proteases, which are involved in the development and processing of embryo development, neurodegeneration, and malignant tumor [8, 9]. It is reported that cell cycle arrest is observed in the majority of cardiomyocytes shortly after birth [10]. In the animal experiments, significant senescent characteristics were observed on the cardiomyocytes isolated from old mice, including elevated SA-β-gal activity, upregulated p53 and p21, accumulated lipofuscin, and declined telomerase activity [11, 12]. p53 is the vital transcriptional factor regulating the fate of the cell. p53-dependent control of p21 expression is one of the key pathways of cell growth arrest and genome integrity. The activation of these factors is the indication of cellular senescence [13]. Aging is reported to be an important element that induces cardiovascular diseases and cardiac hypertrophy is widely observed in the hearts of aged animals or human beings [12, 14]. In addition, similar characteristics are observed in cellular senescence and cardiac hypertrophy, such as the increased size of cardiomyocytes and activated synthesis of proteins [15, 16]. Therefore, targeting the cell senescence in cardiomyocytes might be an effective method for the clinical treatment of pathological cardiac hypertrophy. Arginine vasopressin (AVP) is a hormone that regulates osmotic homeostasis and vasoconstriction in the cardiovascular system. The increased AVP concentration in plasma is positively associated with heart failure [17]. AVP-stimulated cardiomyocytes have been widely used as a cardiomyocyte hypertrophy model in vitro [18]. Metoprolol (Fig. 1) is a β1 receptor blocker used for the clinical treatment of hypertension, stenocardia, and arrhythmia. It was developed by AstraZeneca and approved by the U.S Food and Drug Administration in 1975 [19]. By blocking the β1 receptor located on the membranes of cardiomyocytes, Metoprolol opposes the excitatory effects at the cardiac level, thus decreasing blood pressure by lowering both heart rate and systolic output. With this anti-hypertensive property, it can also counterbalance an increase of the peripheral vascular resistance mainly dependent on the sympathetic innervation of small arterial myocytes [20]. Recently, Metoprolol showed significant protective effects on injured cardiomyocytes [21–24]. In the present study, we investigated the protective effect of Metoprolol against arginine vasopressin-induced cell senescence to explore its potential therapeutic property of Metoprolol on pathological cardiac hypertrophy.

## Materials and Methods

### Cell Culture and Treatments

H9C2 cardiomyocytes were obtained from the Cell Bank of the Chinese Academy of Sciences (Shanghai, China) and cultured in Dulbecco's modified eagle medium (DMEM) supplemented with 10% fetal bovine serum (FBS) at 37 °C and 5% CO_2_. To detect the cytotoxicity of Metoprolol in H9C2 cardiomyocytes, cells were stimulated with 0.5, 1, 5, 10, 50, 100, and 500 μM for 24 h. To determine the protective effects of Metoprolol, cells were stimulated with AVP (10 μM) in the presence or absence of Metoprolol (5, 10 μM) for 24 h.

### Lactate Dehydrogenase (LDH) Release Assay

Briefly, the treated H9C2 cardiomyocytes were planted in a 96-well plate, followed by collecting the supernatant for LDH release assay. The supernatant was incubated with LDH reagents (Jiangcheng, Nanjing, China) for 1.5 h, followed by detecting the absorbance at 450 nm using a Bio-Rad 680 Microplate Reader.

### Cell Counting Kit-8 (CCK-8) Assay

A CCK-8 assay kit (Jiangcheng, Nanjing, China) was used to detect the cell viability of H9C2 cardiomyocytes treated with different strategies according to the instructions of the manufacturer. In brief, the cells were seeded in a 96-well plate followed by adding 10 μL CCK-8 solution in each well. After incubation at 37 °C for 3 h, the absorbance at 450 nm was detected utilizing a PerkinElmer microplate reader (PerkinElmer, Massachusetts, USA).

### Enzyme-Linked Immunosorbent Assay (ELISA)

The concentration of 8OHdG released by the treated H9C2 cardiomyocytes was detected using ELISA assay. Briefly, the supernatants were collected following centrifugation and added to the 96-well plate. After removing the non-specific binding proteins by incubation with 1% bovine serum albumin (BSA) for 1 h, the sample was supplemented with the primary antibodies and incubated at room temperature for 1 h. Subsequently, the plates were washed with PBS buffer and added with streptavidin–horseradish peroxidase (HRP)-conjugated secondary antibodies, followed by being incubated for 20 min at room temperature. Lastly, the microplate spectrophotometer (Thermo, Massachusetts, USA) was used to measure the absorbance at 450 nm.

### SA-β-gal Staining Assay

The treated H9C2 cardiomyocytes were fixed with 2% formaldehyde and 0.2% glutaraldehyde for 10 min at room temperature and then washed using PBS buffer and SA-β-gal staining solution was added (Cell signaling Technologies, Massachusetts, USA), which consisted of 40 mM citric acid/sodium phosphate (pH 6.0), 150 mM NaCl, 2 mM MgCl_2_, 5 mM potassium ferrocyanide, 5 mM potassium ferricyanide, and 1 mg/mL of 5-bromo-4-chloro-3-indoyl-β-d-galactopyranoside. After incubation at 37 °C for 16 h, the percentage of SA-β-gal-positive cardiomyocytes was calculated under a microscope (Olympus, Tokyo, Japan).

### Telomerase Activity

After lysing the treated H9C2 cardiomyocytes using RIPA lysis buffer (Beyotime, Shanghai, China), the supernatant was collected after centrifugation. The telomerase activity was detected utilizing the telomerase ELISA kit (Mlbio, Shanghai, China) according to the description reported previously [25].

### iNampt Activity

The iNampt activity in the cardiomyocytes was detected using the NAMPT Activity Assay kit (Abcam, Cambridge, UK) according to the instructions of the manufacturer. In brief, the lysis of the treated H9C2 cardiomyocytes was mixed with two-step reaction mix 1 and incubated for 60 min at 30 °C. After adding the two-step reaction mix 2, the samples were incubated for 30 min at 30 °C. Lastly, the absorbance at 450 nm was detected using the PerkinElmer microplate reader (PerkinElmer, Massachusetts, USA).

### Measurement of NAD^+^/NADPH Ratio

The NAD^+^/NADPH quantification kit (Bio Vision, San Francisco, USA) was used to detect the cellular concentration of NAD^+^ according to the instructions of the manufacturer. Using the commercial kit, the concentrations of NAD^+^ and NADPH were detected based on an enzyme cycling reaction, a sensitive method for the measurement of NAD^+^ and NADPH levels.

### Sirt1 Activity

The cardiomyocytes were homogenized in lysis buffer containing Tris–HCl, NaCl, phenylmethylsulfonyl fluoride, leupeptin, and aprotinin and then the total proteins were collected after centrifugation for 10 min at 15,000 rpm and 4 °C. The histone deacetylase colorimetric assay kit (Enzo Life Science, New York, USA) was utilized to detect the Sirt1 activity according to the instructions of the manufacturer.

### Immunoprecipitation of Acetylated p53

Cardiomyocytes were lysed and the protein concentration was evaluated using the bicinchoninic acid (BCA) method. 1 mg total protein was mixed with 5 µg anti-acetylated-lysine antibody (CST, Massachusetts, USA) and incubated at 4 °C overnight to form an antigen–antibody complex. 50 µL magnetic beads were then added to the complex solution and incubated at 4 °C for 4 h. After centrifugation by 2500 rmp at 4 °C, the complex was collected and stored at − 80 °C for subsequent western blot analysis using p53 antibody.

### Real-Time PCR Assay

The total RNA was isolated from the treated cardiomyocytes using the Trizol reagents (Thermo, Massachusetts, USA). The extracted RNA was treated with DNase I (Thermo, Massachusetts, USA) for 10 min to remove contaminated cDNA. The quality and concentration of RNA were validated using a Nanodrop 2000 spectrophotometer (Thermo, Massachusetts, USA). The wavelength absorption ratio (260/280 nm) was between 1.8 and 2.0 for all preparations. A total 1 µg of RNA was transcribed into cDNA utilizing the Reliance Select cDNA Synthesis Kit (Bio-Rad, California, USA). In the present study, the PCR reaction was conducted utilizing the SYBR Master Mix kit with a 25-μL reaction system and the StepOne-Plus system (Takara, Tokyo, Japan) according to the following procedure: denaturing at 95 °C for 30 s, annealing at 60 °C for 1 min and extending at 95 °C for 5 s for 40 cycles, and 72 °C 10 min, 1 cycle. Finally, the 2^−ΔΔCt^ method was used to determine the relative expression level of target genes with β-actin used for normalization. The following primers were used in this study: p21, (F: 5′ -TGTTCCACACAGGAGCAAAG-3′, R: 5′-AACACGCTCCCAGACGTAGT-3′), β-actin (F: 5′-CTGCCCTGGCTCCTAGCAC-3′, R: 5′- CGGACGCAGCTCAGTAACAGTCCG-3′).

### Western Blot Assay

After extracting the total proteins from the treated cardiomyocytes with the RIPA Lysis Buffer (Beyotime, Shanghai, China), the proteins were quantified with a BCA kit (Beyotime, Shanghai, China), followed by being loaded and separated by the SDS-PAGE. Then, the separated proteins were transferred to the PVDF membranes (Beyotime, Shanghai, China), followed by incubation with 5% BSA for the removal of non-specific binding proteins. Subsequently, the membrane was incubated with the primary antibody against p53 (1:1000, CST, Massachusetts, USA), p21 (1:1000, CST, Massachusetts, USA), and β-actin (1:1000, Massachusetts, Boston, USA) at 4 °C overnight, followed by being incubated with secondary antibody at room temperature for 1.5 h. Finally, the membrane was incubated with ECL solution (Beyotime, Shanghai, China) and then exposed to Tanon 5200-multi (Tanon, Shanghai, China). ImageJ software was used to quantify the relative expression level of target proteins.

### Data Analysis and Statistical Methods

Data are expressed as mean ± standard errors (S.E.). Statistical analysis was performed using the software SPSS 22.0. All data were tested for normal distribution using the Shapiro–Wilk test. Data with normal distributions were compared using a two-way analysis of variance (ANOVA) followed by a Tukey’s post hoc test. Nonparametric data were analyzed with the Kruskal–Wallis H test. *P* < 0.05 was considered statistically significant.

## Results

### Cytotoxicity of Metoprolol in H9C2 Cardiomyocytes

**Fig. 2:**
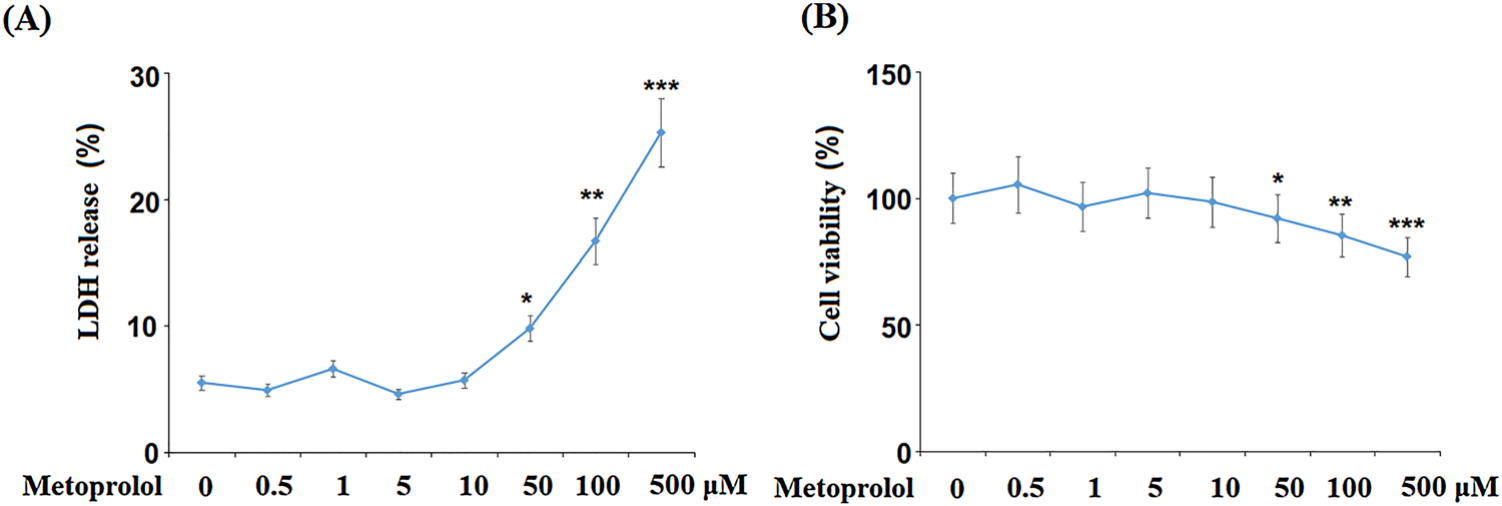
Cytotoxicity of Metoprolol in H9C2 cardiomyocytes. Cells were stimulated with 0.5, 1, 5, 10, 50, 100, and 500 μM for 24 h. **A** LDH release; **B** cell viability (*, **, ***P < 0.05, 0.01, 0.005 vs. control, *n* = 5–6)

To obtain the optimized concentration of Metoprolol for the treatment of H9C2 cardiomyocytes, cells were stimulated with 0.5, 1, 5, 10, 50, 100, and 500 μM for 24 h followed by detecting cytotoxicity using CCK-8 assay and LDH release assays. As shown in Fig. 2A, as the concentration of Metoprolol increased from 0.5 to 10 μM, the LDH release was maintained around 5%, which was then significantly elevated as the concentration of Metoprolol increased to 50 μM. In addition, compared to the control, as the concentration of Metoprolol increased from 0.5 to 10 μM, no significance was observed on the cell viability of the H9C2 cardiomyocytes. It was later dramatically declined to 92.1%, 85.3%, and 76.9% by the treatment with 50, 100, and 500 μM Metoprolol, respectively (Fig. 2B). Therefore, in the subsequent experiments, 5 and 10 μM Metoprolol were used.

### Metoprolol Ameliorated Arginine Vasopressin (AVP)-Induced Oxidative Stress in H9C2 Cardiomyocytes

**Fig. 3:**
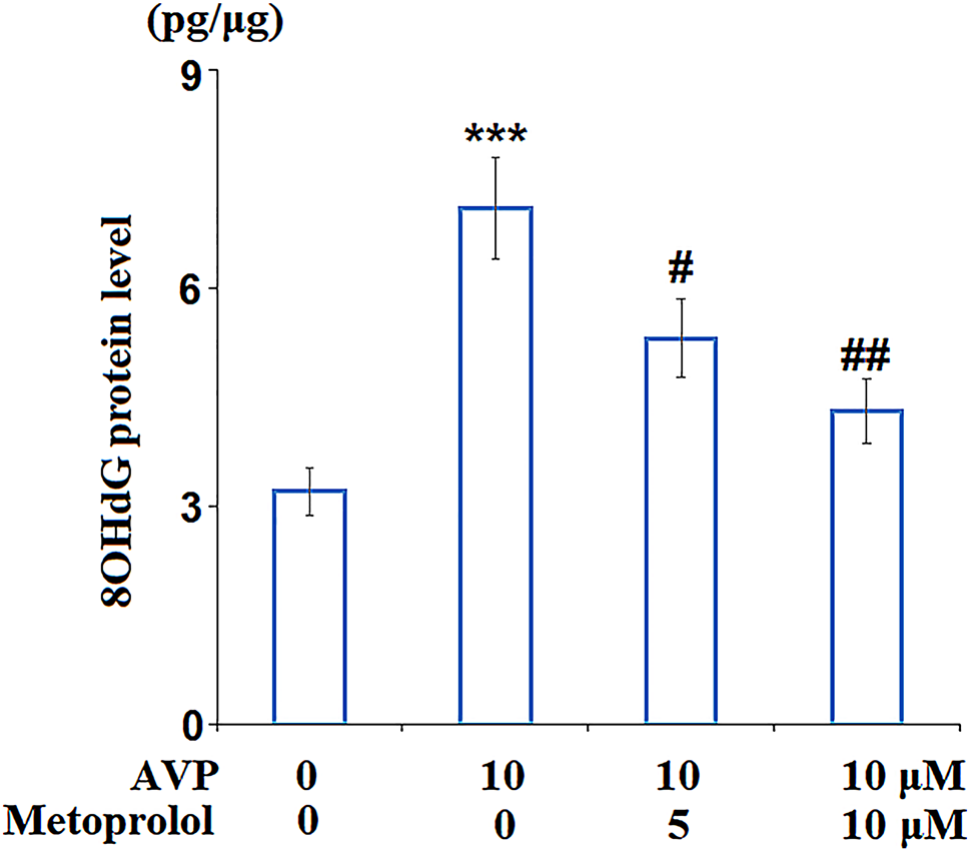
Metoprolol ameliorated arginine vasopressin (AVP)-induced oxidative stress in H9C2 cardiomyocytes. Cells were stimulated with AVP (10 μM) in the presence or absence of Metoprolol (5, 10 μM) for 24 h. Levels of 8OHdG were measured using ELISA (***P < 0.005 vs. control; #, ##*P* < 0.05, 0.01 vs. AVP group, *n* = 5–6)

As shown in Fig. 3, compared to the control group, the level of 8OHdG was elevated from 3.2 to 7.1 pg/μg DNA by stimulation with AVP. It was then suppressed to 5.3 and 4.3 pg/μg DNA by treatment with 5 and 10 μM Metoprolol, respectively, indicating the oxidative stress in cardiomyocytes induced by AVP was significantly ameliorated by Metoprolol.

### Metoprolol Ameliorated AVP-Induced Cell Senescence

**Fig. 4:**
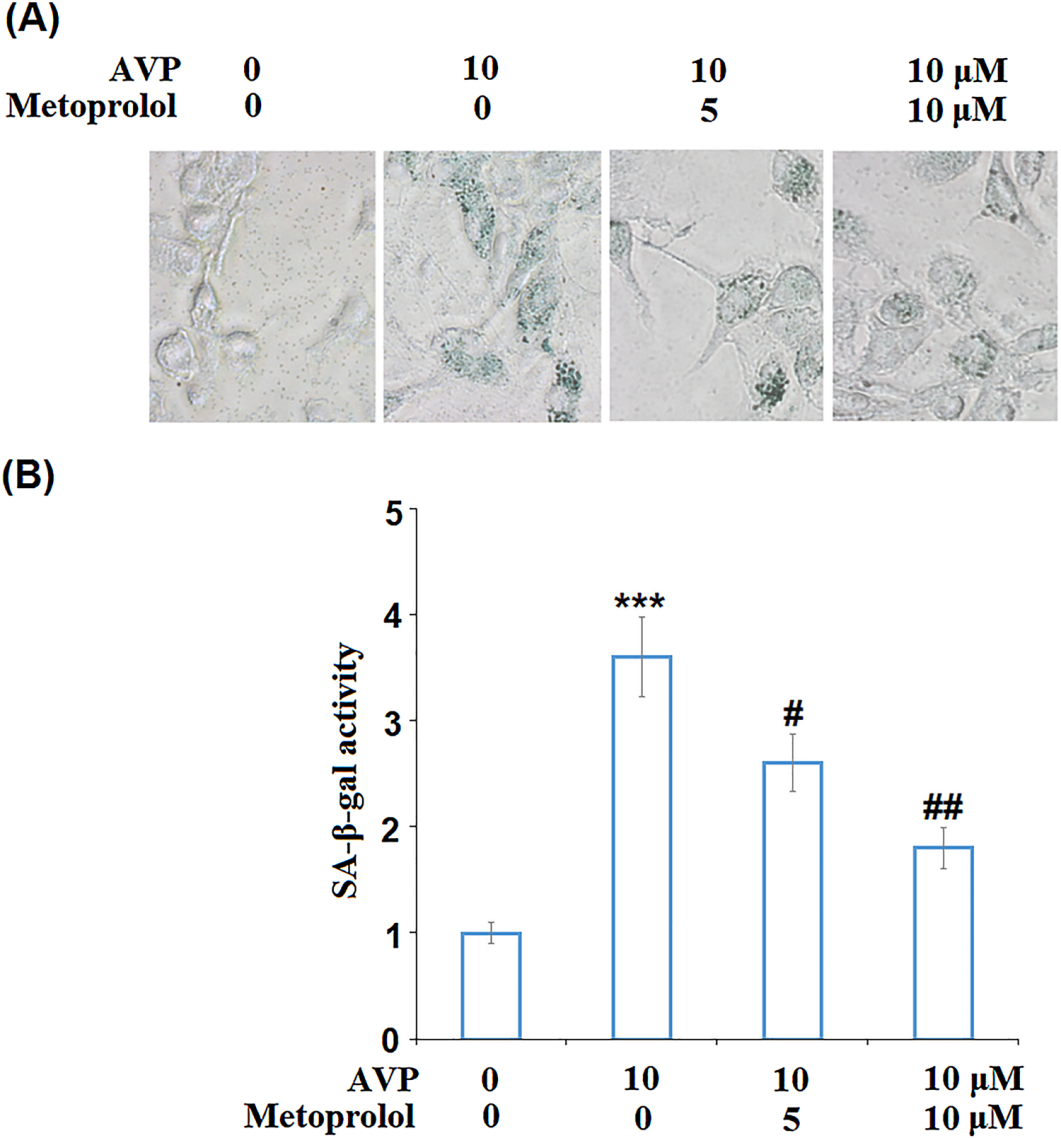
Metoprolol ameliorated arginine vasopressin (AVP)-induced cell senescence. Cells were stimulated with AVP (10 μM) in the presence or absence of Metoprolol for 7 days. **A** Senescence-associated beta-galactosidase (SA-β-gal) staining; **B** quantification of SA-β-gal activity (***P < 0.005 vs. control; #, ##*P* < 0.05, 0.01 vs. AVP group, *n* = 5–6)

We further investigated the impact of Metoprolol on cell senescence in AVP-challenged cells. Representative SA-β-gal staining results are shown in Fig. 4A. The percentage of SA-β-gal-positive cells was significantly elevated by stimulation with AVP. Interestingly, the introduction of Metoprolol dose-dependently decreased this percentage (Fig. 4B), indicating that the cell senescence induced by AVP was significantly alleviated by Metoprolol.

### Metoprolol Ameliorated AVP-Induced Decrease in Telomerase Activity

**Fig. 5:**
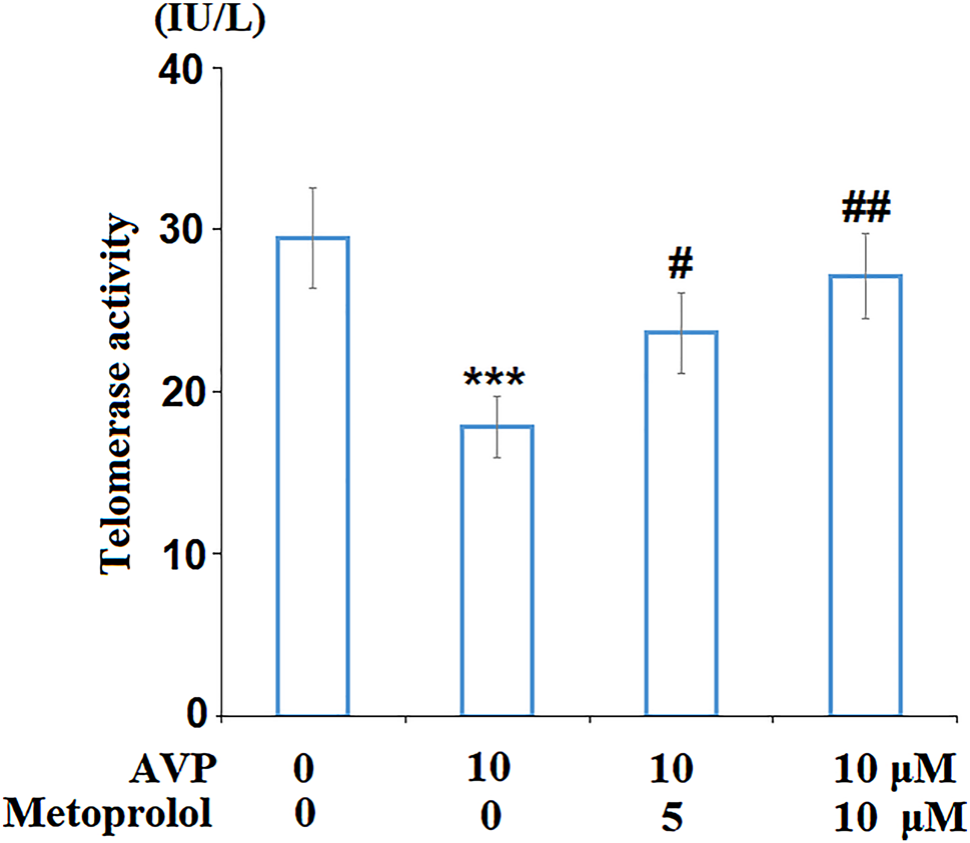
Metoprolol ameliorated arginine vasopressin (AVP)-induced decrease in telomerase activity. Cells were stimulated with AVP (10 μM) in the presence or absence of Metoprolol for 7 days. Telomerase activity was assessed (***P < 0.005 vs. control; #, ##*P* < 0.05, 0.01 vs. AVP group, *n* = 5–6)

Cells were stimulated with AVP (10 μM) in the presence or absence of Metoprolol (5 and 10 μM) for 7 days. As shown in Fig. 5, compared to the control group, the telomerase activity was decreased from 29.5 to 17.8 IU/L by the stimulation with AVP but pronouncedly elevated to 23.6 and 27.1 IU/L by the introduction of Metoprolol in a dose-dependent manner.

### Metoprolol Rescued AVP-Induced Dysfunction in the iNampt–NAD^+^–SIRT1 System

**Fig. 6:**
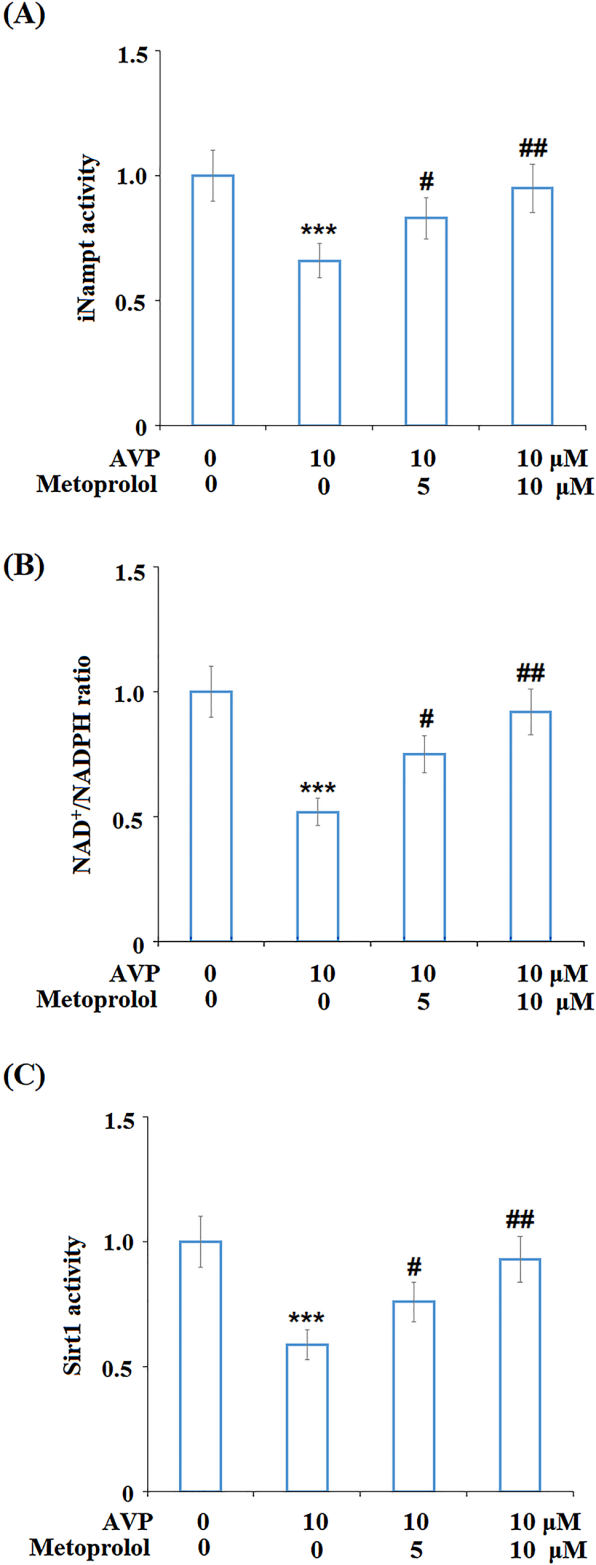
Metoprolol rescued Arginine vasopressin (AVP)-induced dysfunction in the iNampt–NAD^+^–SIRT1 system. Cells were stimulated with AVP (10 μM) in the presence or absence of Metoprolol for 24 h. **A** iNampt activity; **B** NAD^+^/NADPH ratio; **C** Sirt1 activity (***P < 0.005 vs. control; #, ##*P* < 0.05, 0.01 vs. AVP group, *n* = 5–6)

To explore the effects of Metoprolol on the normal function of cardiomyocytes, cells were stimulated with AVP (10 μM) in the presence or absence of Metoprolol (5 and 10 μM) for 24 h, and the iNampt activity, NAD^+^/NADPH ratio, and Sirt1 activity were examined using commercial kits. As shown in Fig. 6A–C, compared to the control, AVP suppressed the iNampt activity, NAD^+^/NADPH ratio, and Sirt1 activity, all of which were elevated by treatment with Metoprolol in a dose-dependent manner.

### Metoprolol Mitigated AVP-Induced Increase in Acetylation of p53 and the Expression of p21

**Fig. 7:**
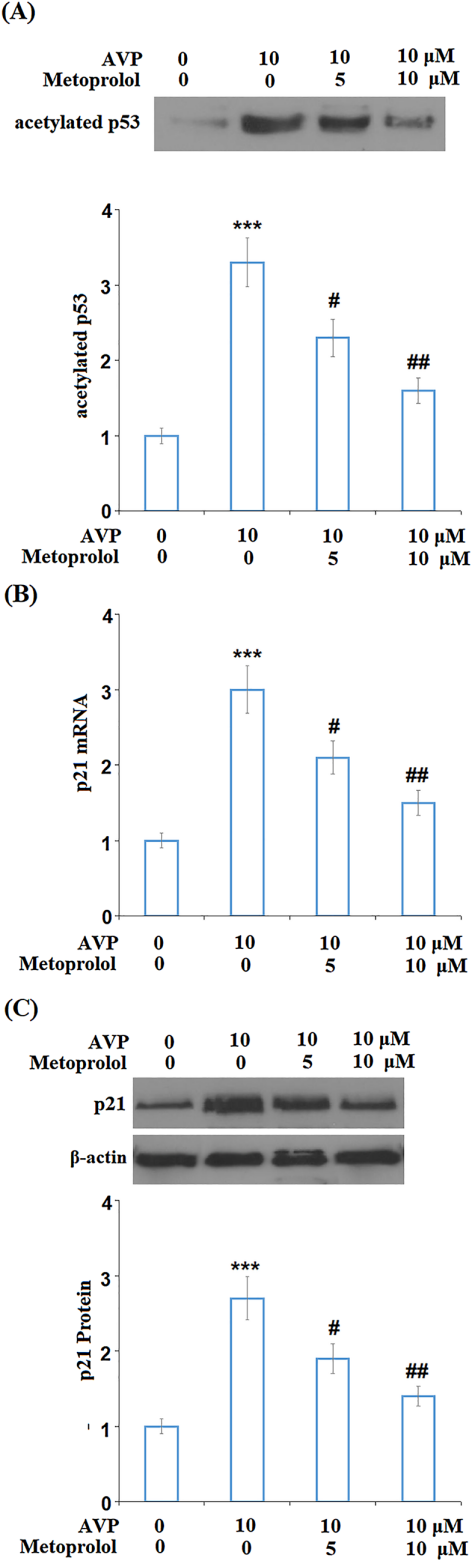
Metoprolol mitigated Arginine vasopressin (AVP)-induced increase in acetylation of p53 and the expression of p21. Cells were stimulated with AVP (10 μM) in the presence or absence of Metoprolol for 24 h. **A** Levels of acetylated p53 were measured with immunoprecipitation; **B** mRNA of p21; **C** protein of p21 (***P < 0.005 vs. control; #, ##*P* < 0.05, 0.01 vs. AVP group, *n* = 5)

CDKIs, such as p53 and p21, are important biomarkers of cell senescence, the expression levels of which were detected following the treatments. Sirt1 acts as an inhibitor of p53 acetylation. Interestingly, we found that the upregulated acetylated p53 in the AVP group was significantly downregulated by the introduction of Metoprolol (Fig. 7A). In addition, as shown in Fig. 7B, C, compared to the control group, AVP significantly promoted the expression level of p21, which was then inhibited by treatment with Metoprolol in a dose-dependent manner at both the mRNA and protein levels.

## Discussion

Cell senescence is observed in the cardiac hypertrophy model induced by angiotensin II and in dilated cardiomyopathy caused by the silencing of Bmi1, which is verified by the elevated proportion of SA-β-gal-positive cells [26]. Oxidative stress is an important inducer for cell senescence [27]. The production of reactive oxygen species (ROS) is involved in cardiac remodeling following isoproterenol injection, including wall stiffness and fibrogenesis [28, 29]. In addition, the inhibition of myocyte autophagy was observed in cardiac hypertrophy, proven to be closely related to the activation of oxidative stress [30, 31]. The accumulation of lipofuscin is induced by the activated oxidative stress and suppressed autophagy, which is believed to be an important inducer for cardiac dysfunction in aged rats [11, 12]. AVP is a nonapeptide secreted by the posterior pituitary gland and hypothalamus. It stimulates vasoconstriction by regulating the function of the adenosine triphosphate-sensitive potassium channel and inducing the production of nitric oxide [32], which is proven to be closely related to the development of heart failure [33, 34]. In the present study, AVP was used to induce the in vitro injury model on cardiomyocytes, which was verified by the activated oxidative stress and aggravated cell senescence. The state of oxidative stress and cell senescence were dramatically alleviated by treatment with Metoprolol, indicating its protective effect on the injury of cardiomyocytes induced by AVP. In our future work, more evidence will be provided to further confirm the protective effects of Metoprolol, such as detecting the level of mitochondrial ROS, measuring the production of inflammatory factors, and determining the activity of superoxide dismutase (SOD).

Sirtuin 1 (Sirt1) is reported to be a NAD^+^-dependent deacetylase and plays an important role in regulating cell senescence, apoptosis, and the cell cycle by mediating the acetylation of several transcriptional factors, including p53, FOXO, and histones [35, 36]. It is reported that the concentration of NAD^+^ could be inhibited by the activation of nicotinamide phosphoribosyltransferase (iNampt), which is a rate-limiting enzyme for the synthesis of NAD^+^ and it inhibits the activity of Sirt1 [37, 38]. Therefore, Sirt1 is an important regulatory transcriptional factor that negatively regulates the development and processing of cell senescence induced by oxidative stress [39] by deacetylating p53. In the present study, we found that Metoprolol rescued the significantly reduced activities of Sirt1 and iNampt, as well as the concentration of NAD by AVP. These data verify the protective effects of Metoprolol on the AVP-induced cell senescence in cardiomyocytes.

Protein p53 is a tumor suppressor comprised of 393 amino acids and is an important transcriptional factor that regulates the cell cycle, cell senescence, and apoptosis. Its upregulation induces cardiomyocytes cell death by suppressing the angiogenesis in the heart tissues and its silencing of p53 is reported to alleviate the heart function [40]. Acetylated p53 plays an important role in mediating the expression of its downstream genes such as p21.

Our data show that AVP suppressed SIRT-1 activity and increased acetylated p53 and released p21 expression. The acetylation of p53 and its induction on its downstream target p21 has been shown to contribute to genomic instability and cellular senescence [41]. In the cardiovascular system, SIRT-1/p53 has been shown to modulate cardiovascular aging [42]. In our experiment, AVP treatment reduced iNampt activity, which is the rate-limiting enzyme in the process of NAD + production. As a result, cellular NAD + /NADPH was reduced (Fig. 6B). SIRT-1 is an NAD + -dependent deacetylase, and its activity was decreased when the availability of NAD + was reduced (Fig. 6C). However, the addition of Metoprolol ameliorated AVP-elicited effects. Therefore, the action of Metoprolol on the NAD + pathway could be responsible for its effect on SIRT-1/p53.

In summary, our data demonstrate the protective effects of Metoprolol on AVP-induced cardiomyocyte senescence. Mechanistically, we show that Metoprolol protected cellular senescence by regulating the Sirt1/p53/p21 axis. Our study provides new evidence on the β1 blocker Metoprolol in cardiac protection.

## Data Availability

The data that support the findings of this study are available from the corresponding author upon reasonable request.
